# The safety and efficacy of inhaled dry powder mannitol as a bronchial provocation test for airway hyperresponsiveness: a phase 3 comparison study with hypertonic (4.5%) saline

**DOI:** 10.1186/1465-9921-6-144

**Published:** 2005-12-09

**Authors:** John D Brannan, Sandra D Anderson, Clare P Perry, Ruth Freed-Martens, Anna R Lassig, Brett Charlton, the Aridol Study Group

**Affiliations:** 1 Department of Respiratory Medicine, 11 West, Royal Prince Alfred Hospital, Missenden Road, Camperdown NSW 2050, Australia; 2 Pharmaxis Ltd., Unit 2, 10 Rodborough Rd, Frenchs Forest NSW 2086, Australia

## Abstract

**Background:**

Inhaled mannitol is a new bronchial provocation test (BPT) developed to improve portability and standardisation of osmotic challenge testing. Osmotic challenge tests have an advantage over the traditional methods of measuring airway hyperresponsiveness using methacholine as they demonstrate higher specificity to identify asthma and thus the need for treatment with inhaled corticosteroids (ICS). The safety and the efficacy of mannitol (M) as a BPT to measure airway hyperresponsiveness were compared to hypertonic (4.5%) saline (HS) in people both with and without signs and symptoms of asthma.

**Methods:**

A phase III, multi-centre, open label, operator-blinded, crossover design, randomised trial, with follow-up. Asthmatics and non-asthmatics (6–83 yr) were recruited and 592 subjects completed the study. Mannitol was delivered using a low resistance dry powder inhaler and HS was delivered using an ultrasonic nebuliser. The FEV_1 _was measured 60 seconds after each dose of mannitol (5,10,20,40,80,160,160,160 mg) and after each exposure to HS (0.5,1.0,2.0,4.0,8.0 minutes). A 15% fall in FEV_1 _defined a positive test. Adverse events were monitored and diaries kept for 7 days following the tests.

**Results:**

Mean pre-test FEV1 (mean ± SD) was 95.5 ± 14% predicted. 296 were positive to mannitol (M+) and 322 positive to HS (HS+). A post study physician conducted clinical assessment identified 82.3% asthmatic (44% classified mild) and 17.7% non-asthmatic. Of those M+, 70.1% were taking ICS and of those mannitol negative (M-), 81.1 % were taking ICS. The % fall in FEV1 for mannitol in asthmatics was 21.0% ± 5.7 and for the non-asthmatics, 5.5% ± 4.8. The median PD15 M was 148 mg and PD15 HS 6.2 ml. The sensitivity of M to identify HS+ was 80.7% and the specificity 86.7%. The sensitivity of M compared with the clinical assessment was 59.8% and specificity 95.2% and increased to 88.7% and 95.0% respectively when the M- subjects taking ICS were excluded. Cough was common during testing. There were no serious adverse events. The diarised events were similar for mannitol and HS, the most common being headache (17.2%M, 19%HS), pharyngolaryngeal pain (5.1%M, 3%HS), nausea (4.3%M, 3%HS), and cough (2.2%M, 2.4%HS).

**Conclusion:**

The efficacy and safety of mannitol was demonstrated in non-asthmatic and clinically diagnosed asthmatic adults and children.

## Background

The bronchial provocation test that uses an aerosol of hypertonic (4.5%) saline as an indirect stimulus was developed over a number of years by Anderson and others in Australia [1-4]. The test subsequently became known as the hypertonic saline (HS) test. In epidemiology it was first used in a study in children with exercise as a comparator challenge [5]. The HS test has now been used safely in both adults and children for a wide variety of studies for 20 years [6-9]. A 15% reduction in forced expiratory volume in one second (FEV_1_) is used to define abnormality [10]. The dose of 4.5% saline to provoke this fall in FEV_1 _(PD_15_) is the index used to express the airway sensitivity to this stimulus or the airway hyperresponsiveness (AHR) [10]. There are some technical difficulties with the HS test that preclude it from being a common operating standard for investigators [4].

In order to overcome some of these problems a dry powder preparation of mannitol was developed to provide a more simple form of osmotic challenge [11]. The studies using dry powder mannitol demonstrated that it had a very similar profile to HS [12]. Further those subjects responsive to other challenge tests that acted 'indirectly' to cause airways to narrow e.g. exercise, eucapnic voluntary hyperpnea [13], and adenosine monophosphate [14], were also responsive to mannitol. The major advantage in using provoking stimuli that act 'indirectly' to cause airways to narrow is the high specificity for identifying the type of AHR that is altered by drugs used in the treatment of asthma [15,16], and inhaled corticosteroids in particular. Sensitivity to mannitol is reduced or even totally inhibited following chronic administration of inhaled corticosteroids (ICS) [17], therefore allowing monitoring of therapy [18]. Further, in subjects whose asthma is well controlled with ICS, response to mannitol has been used to predict exacerbations following back titration of steroid dose [19]. In these respects challenge with mannitol is different from challenge with methacholine. Whilst responsiveness to methacholine can be reduced 1 to 2 doubling doses in response to treatment with inhaled steroids, most patients still record a positive response [20-22] and this agent has not been useful to guide for titration of steroid dose [23].

We aimed to determine the efficacy and safety profile of a commercial preparation of dry powder mannitol in subjects with and without signs and symptoms of asthma. A phase III, multi-centre, open label, operator-blinded, crossover design, randomised trial, with follow-up was performed comparing the airway response to mannitol with the HS test and a standard clinical assessment of asthma.

## Methods

### Study design

The study was an operator blinded, randomised, crossover comparator study. At Visit 1, after entry and randomisation to determine the order of each challenge, each subject answered a questionnaire, had skin prick tests for aeroallergens then underwent the first challenge. The second challenge was scheduled one week later at Visit 2 and the study was complete a week after the second challenge at Visit 3, when each subject returned for a measurement of spirometry. The respiratory scientist conducting the initial challenge was blinded to the subject's asthma status and the respiratory scientist conducting the reciprocal challenge was blinded to the results of the initial challenge and the asthma status of the subject. The respiratory physician was blinded to the results of the mannitol challenge and the identity of the subject when determining the asthma status of the subject.

The protocol was approved by the Ethics Review Committee of each participating hospital. The trial was carried out under the Clinical Trial Notification scheme of the Therapeutic Goods Administration of Australia (CTN Nos 2003/240, 035/2003).

### Subjects

**Figure 1:**
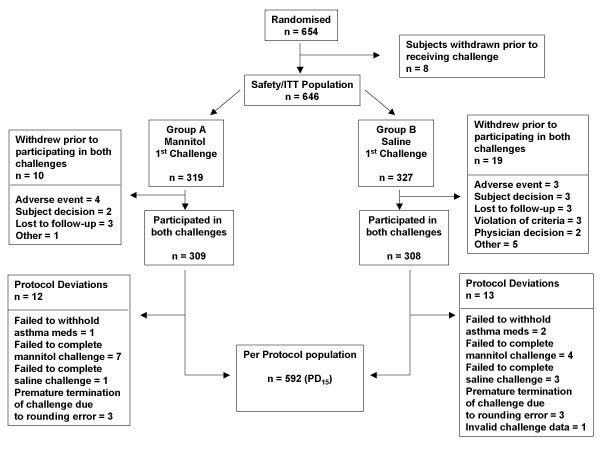
Flow chart showing the progression of subjects through the study.

Of six hundred and fifty-four subjects 6 years of age and above recruited from the local community and from pulmonary function clinics, 646 completed at least one challenge and were included in the population that could be analysed for the safety analysis (Table 1) (Figure 1). Of these, 592 subjects (466 adults, 126 children 6–83 yrs) completed both challenge tests and were used for the efficacy analysis.

**Table 1:** Demographics of populations

	**Safety Population (n = 646)**	**Efficacy Population (n = 592)**
Age (yrs)	34.8 (6 to 83)	34.7 (6 to 83)
Gender	47% M: 53% F	46% M: 54% F
Race	91% Caucasian	91% Caucasian
BMI (kg/m^2^)	25.5 (0.6)	25.5 (0.6)
Height (cm)	164.2 (14.0)	164.3 (13.9)
Weight (kg)	70.2 (21.6)	70.1 (21.6)
Asthmatics (n)	551	505
FEV_1 _(L)	3.0 (0.9)	3.0 (0.9)
% Predicted FEV_1_	95.0 (14.5)	95.5 (14.7)
Non-asthmatics (n)	95	91
FEV_1 _(L)	3.2 (0.9)	3.2 (0.9)
% Predicted FEV_1_	94.6 (14.1)	95.2 (14.4)

The asthmatic subjects were required to have active signs and symptoms of asthma according to the National Asthma Council of Australia Asthma Management Handbook Guidelines [24]. The non-asthmatic subjects were required never to have had a clinical diagnosis of asthma or experienced signs and symptoms suggestive of asthma. All subjects gave informed consent to participate in this study in accordance with ICH GCP guidelines and local regulatory requirements.

Subjects were required to have a baseline FEV_1 _greater than 70% of normal predicted values for asthmatic subjects OR greater than 80% of normal predicted values for non-asthmatic subjects. No subject was to have an active upper or lower respiratory tract infection severe enough to require a medical consultation or any other acute or chronic pulmonary disorder including:- cystic fibrosis, chronic obstructive pulmonary disease, bronchiectasis, chronic bronchitis, emphysema, tuberculosis, and carcinoma. Further, subjects with current uncontrolled hypertension or known aortic aneurysm, myocardial infarction or cerebral vascular accident in the last six months, or ocular or abdominal surgery in the three months prior to enrolment were excluded from the study.

No subject could be breast feeding or pregnant, or have participated in another trial in the previous 4 weeks, be related to a study investigator, or have a known intolerance to mannitol or salbutamol. No subject was to have a previous admission to Intensive Care for asthma in the two years prior to study entry, and no subject could be taking oral / parenteral corticosteroids in the two weeks prior to study enrolment.

All subject were required to be able to perform repeatable spirometry according to American Thoracic Society (ATS) criteria [25], and to withhold strenuous exercise and smoking for 6 hr; caffeine, short acting bronchodilators (SABA), sodium cromoglycate and nedocromil sodium for 8 hr; inhaled corticosteroids (ICS) alone and combination with long acting bronchodilators (LABA), and short acting anti-cholinergics for 12 hr; theophylline for 24 hr; long acting anti-cholinergics and anti-histamines for 72 hr; and leukotriene antagonists for 4 days. Subjects were encouraged to keep their dose of inhaled corticosteroids constant over the course of the study. Visit 2 pre-challenge FEV_1 _was required to be within 10% of that at Visit 1. Challenges were performed at the same time of day ± 2 hours.

### Questionnaires

Two questionnaires were carried out, one investigating respiratory history over the preceding 12 months and based on the International Study of Asthma and Allergies in Childhood (ISAAC) Steering Committee Phase 1 and 2 modules [26]. The other was an investigator designed questionnaire about symptoms for the week immediately prior to each visit. The symptom questions included frequency and severity of cough (during the day or night) and wheeze, bronchodilator use, number of days troubled by breathing and number of days that symptoms interfered with work or school activities.

Each subject was issued with a study diary at Visit 1 and requested to complete the diary every day up to their third visit when they handed it to the investigator. The diary was a record of adverse events, respiratory symptoms and concomitant medications. The investigator was responsible for reviewing the diary for completeness at the final visit.

### Mannitol challenge

Dry powdered mannitol (Aridol™) was supplied in kit form (Pharmaxis Ltd., NSW Australia) and contained one empty capsule (0 mg), 2 × 5, 2 × 10, 2 × 20, and 18 × 40 mg capsules. The dry powder device used for inhalation was the Osmohaler™ (RS-01, Plastiape™, Italy), a single capsule device with a low inspiratory resistance. The mannitol challenge required the FEV_1 _to be measured 60 seconds after each mannitol dose (5,10,20,40,80,160,160,160 mg). If the FEV_1 _fell by = 10% on any one dose then that same dose was repeated.

The challenge started with the empty capsule. This was loaded into the device and punctured by the investigator. The subjects were asked to inhale from the device from near to functional residual capacity to near to total lung capacity and to hold their breath for 5 seconds. Subjects were encouraged to keep a nose clip on for 10 seconds after inhalation and then exhale through their mouth to minimise deposition in the nasopharynx. In addition to providing the baseline FEV_1_, the inclusion of the 0 mg capsule demonstrated the sound and use of the device to the subject. Hearing the capsule rotating indicated that sufficient inspiratory flow had been achieved and that it was positioned correctly in the chamber. Sixty seconds after inhalation of the 0 mg capsule the FEV_1 _was measured and two repeatable values were obtained and recorded. The highest of these values was taken as the baseline FEV_1 _and was used to calculate the target FEV_1 _value that indicated a 15% fall in response to the mannitol challenge. This value was calculated immediately after the administration of the 0 mg capsule.

The first dose of mannitol (5 mg) was administered, and sixty seconds later the FEV_1 _was measured to obtain two repeatable FEV_1 _values. This procedure was repeated for each dose step until a 15% fall in FEV_1 _was achieved or the cumulative dose of 635 mg had been administered. At each dose step, oxygen saturation and cough severity were recorded.

### Hypertonic (4.5%) saline challenge

The initial exposure time for inhaling the 4.5% saline was for 30 sec, then 1 min, 2 min, 4 min, and 8 min. The FEV_1 _was measured at least twice 60 seconds after each exposure time. The subjects wore a nose clip and were encouraged to breathe normally through a two-way non-rebreathing valve. If the fall in FEV_1 _was = 10% on any one exposure time the same exposure time was repeated. The ultrasonic nebuliser was required to have an output of at least 1.5 ml/min. After each exposure time, oxygen saturation and cough severity were recorded.

### Calculation of responses

The highest value of 2 repeatable FEV_1 _measurements made after each dose was used in the calculation for the % fall in FEV_1_. The dose of mannitol (mg) or saline (ml) to provoke a 15% fall in FEV_1 _(PD_15_) was calculated by linear interpolation from the curve relating the % fall in FEV_1_. For mannitol this was from the post 0 mg capsule baseline value for FEV_1 _to the cumulative dose of mannitol delivered (e.g. 5 mg, 15 mg, 35 mg, 75 mg, 155 mg, 315 mg, 475 mg, 635 mg). For saline, the amount of aerosol delivered per minute was calculated by dividing the total amount delivered by the time of delivery (e.g. 28 ml in 15.5 min = 1.81 ml/min). The dose was expressed cumulatively over time.

The response dose ratio (RDR) was calculated by taking the final % fall in FEV_1 _recorded and dividing it by the cumulative dose of mannitol or saline administered to induce that % fall. For calculation of the RDR a value of 0.1 was used for those having 0% fall in FEV_1_.

Efficacy was analysed using sensitivity and specificity of the mannitol challenge with respect to the 4.5% saline challenge and the clinical assessment. Subjects were considered positive to a test if at least a 15% reduction in FEV_1 _from baseline occurred. Subjects who reached the end of a challenge with <15% reduction in FEV_1 _were considered to have a negative response.

### Recovery

On completion of the challenge, subjects with a 15% or more percentage fall in FEV_1 _received 200 mcg of the beta_2 _adrenoceptor agonist salbutamol via a Volumatic™ spacer. If the FEV_1 _had not returned to within 5% of the baseline FEV_1 _in 15 minutes a second dose of salbutamol was administered.

### Vital signs for challenge

Vital Signs measured were sitting blood pressure, oxygen saturation by pulse oximetry (SpO_2_), respiratory rate, and heart rate measured at the beginning of the challenge, on immediate completion of the challenge (before administration of salbutamol in those with a positive response), and 15 minutes after challenge.

### Clinical assessment

All subjects were designated asthmatic or non-asthmatic based on Respiratory History Questionnaire, Respiratory Symptom Questionnaire, spirometry results, concomitant medications and the result of the 4.5% saline challenge. The subjects *were not *interviewed by the Respiratory Physicians who were blinded to the identity of the subjects. Subjects with a diagnosis of asthma were graded by the physician according to the GINA guidelines [27].

### Statistical methods

The sample size of 600 was primarily based on safety and chosen to be large enough to document the adverse event profile of dry powder mannitol. In the total population, at least 100 were required to be aged 6 × 17 years, 10 of these being non- asthmatics. To compare the adverse events for mannitol and HS 2 × 2 contingency tables were used. Efficacy was analysed in terms of response to each challenge (mannitol and saline) in subjects that had both mannitol and HS challenge, and in terms of the investigator's clinical assessment or the classification of asthmatic/symptomatic or non-asthmatic at study entry. The challenge results are represented as: M+/M- = positive/negative test result with mannitol, S+/S- = positive/negative test result with HS, A+/A- = asthmatic-symptomatic/non-asthmatic at study entry, C+/C- = asthmatic/non-asthmatic by physician's clinical diagnosis at Visit 3. Cross-tabulations were performed to create 2 × 2 tables of the number of subjects within each of four cells for each challenge. In the analysis of mannitol vs HS, the four cells comprised of those positive to both challenges (M+, S+), those negative to both challenges (M-, S-), those positive to mannitol and negative to HS (M+, S-), and those negative to mannitol and positive to HS (M-, S+). Sensitivity was calculated as the probability of a positive test result with mannitol, given a positive HS result (Pr(M+ | S+)), i.e. the number of subjects positive to mannitol out of the total number of subjects positive to HS. Specificity was calculated as the probability of a negative test result with mannitol, given a negative HS result (Pr(M- | S-)), i.e. the number of subjects negative to mannitol out of the total number of subjects negative to HS.

A cross over logistic regression analysis was performed using the results of the mannitol and HS challenges to examine sequence effects, and another to look for study site differences. A cross over regression analysis was also performed to investigate differences between mannitol and HS in the change from baseline (0 mg for mannitol, pre challenge for HS) to end of challenge for the vital sign parameters: systolic and diastolic blood pressure, oxygen saturation, heart rate and respiratory rate.

## Results

All the subjects classed as asthmatics at entry had an established history of asthma, with most having symptoms before 19 years of age. Asthmatic subjects had normal FEV_1 _values and there was no difference compared to the non-asthmatics (Table 1). A total of 646 subjects completed at least one challenge with 627 exposed to mannitol and 636 to HS. The mean dose of mannitol administered was 433.6 ± 237.2 mg, with the mean for subjects with a positive mannitol test being 239.0 ± 185.0 mg. As expected, the mean ± SD cumulative dose for a negative mannitol test was 635 ± 0.9 mg. For the HS challenge, the mean dose ± SD for all subjects was 21.6 ± 13.5 ml, 12.9 ± 11.3 ml in subjects with a positive HS challenge and 32.4 ± 6.3 ml for subjects with a negative challenge. The output of the ultrasonic nebuliser ranged from 0.24 to 4.4 ml/min, with a mean of 2.1 ± 0.5 ml/min.

In the efficacy population (n = 592) that included both asthmatics and non-asthmatics, the severity of symptoms using the questionnaire specific to the week prior to commencement of the study revealed that there were 39.0% (n = 231) with wheeze, 32.8% (n = 194) with trouble breathing, and 11.3% (n = 67) who had respiratory symptoms that interfered with normal activities. There were 45.2% (n = 292) who had daytime cough that lasted for a mean ± SD of 4.0 ± 2.5 days, with 82.5% (n = 241) stating that it was only occasional. Cough at night time was reported by 31.6% (n = 187) and 49.5% (n = 293) reported using a reliever puffer in the past week. Only 6.3% (n = 37) reported having a cold or flu in the past week. There were 78.4% taking at least one medication for asthma. Of those assessed as asthmatics at entry 27.7 % were taking ICS alone and 38.5% in combination with a LABA, 15.6% were taking only a SABA and 3 subjects taking only a LABA. Only 1.7% were taking leukotriene antagonists, 4.6% were taking an anti-cholinergic and 2.2% were taking sodium cromoglycate.

As designated by the clinical assessment, 82.3% (n = 487) of the efficacy population (n = 592) were asthmatic and 17.7% (n = 105) were non-asthmatic. Of the 378 adult asthmatic subjects, 50.5% (n = 191) were classed as mild, 39.9% (n = 151) were classed as moderate, and 9.5% (n = 36) as severe (Table 2). For the 109 paediatric asthmatic subjects, 22.0% (n = 24) were classed as infrequent episodic, 17.4% (n = 19) as frequent episodic, and 60.6% (n = 66) were classed as persistent.

**Table 2:** Clinical classification based on GINA guidelines of all asthmatics, including those who returned a positive challenge

**ADULTS**	**Mild**	**Moderate**	**Severe**
n (Total)	191	151	36
n (with PD_15_)	80	107	35
GeoMean PD_15 _mg (95% CI)	186 (148, 233)	78* (58, 106)	56* (36, 98)

CHILDREN	Infrequent Episodic	Frequent Episodic	Persistent

n (Total)	24	19	66
n (with PD_15_)	18	14	54
GeoMean PD_15 _mg (95% CI)	113 (59, 217)	117 (73, 187)	67 (46, 99)

* p < 0.001 compared with mild

**Figure 2:**
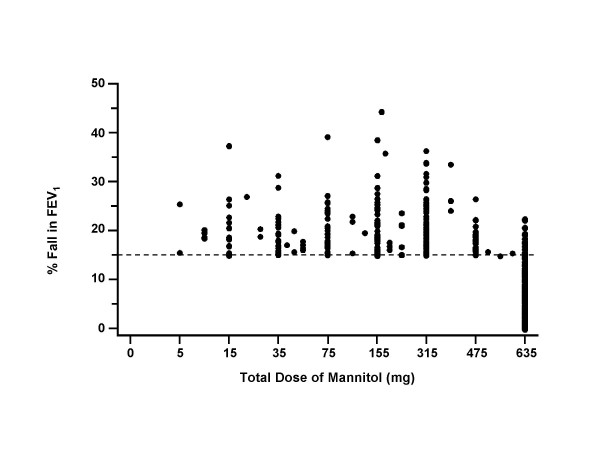
The percent reduction in FEV_1 _from baseline in relation to the total dose of mannitol for the 592 subjects with and without symptoms of asthma of which 292 were positive to the mannitol challenge.

Of the 592 subjects 50.0% (n = 296) were positive to the mannitol challenge and 54.4% (n = 322) positive to HS. For those who were positive the maximum mean % fall in FEV1 was similar for both mannitol (21.0% ± 5.7 (range: 15.0–45.5)) and for HS (21.3% ± 5.9 (range:15.0–48.62)) and there was a relationship between maximum % fall in FEV_1 _to both challenges (r_p _= 0.62, p < 0.001). The maximum mean % fall in FEV_1 _in children less than 12 years of age was 21.1% ± 6.0 (range:15.85 × 40.30) for mannitol and 22.0% ± 6.4 (range:16.34–48.62) for HS. Only 14 subjects recorded a maximum fall in FEV_1 _greater than 30% and the majority had a fall between 15% and 25% (Figure 2). For hypertonic saline 18 subjects recorded a fall of 30% or more.

The geometric mean (95% CI) for PD_15 _was 112.20 mg (97.72 mg, 128.82 mg) for mannitol and 5.01 ml, (4.27 ml, 5.75 ml) for HS. The median PD_15 _for mannitol was 148.1 mg and for HS it was 6.2 ml. There was a moderate, though statistically significant, correlation between log transformed PD_15 _between mannitol and HS (r = 0.53, p < 0.0001). Those adults with a classification of mild asthma had a PD_15 _for mannitol that was significantly different from those classed as having moderate and severe asthma (Table 2). There was a trend for those children with episodic asthma to have higher PD_15 _values however there was no significant difference between the responses and their clinical severity classification.

The maximum mean % fall in FEV_1 _from baseline for subjects who were clinically assessed as non-asthmatic was 5.5% ± 4.8 after mannitol and 5.4% ± 4.9 after HS. The median (range) RDR for those with a negative challenge was 0.0075 (0.0–0.0317) for mannitol and 0.15 (0.0–0.70) for HS. For the group who were non-asthmatic the RDR geometric mean (95% CI) for mannitol was 0.0029 (0.002, 0.004) and for the HS was 0.11 (0.085, 0.139).

**Figure 3:**
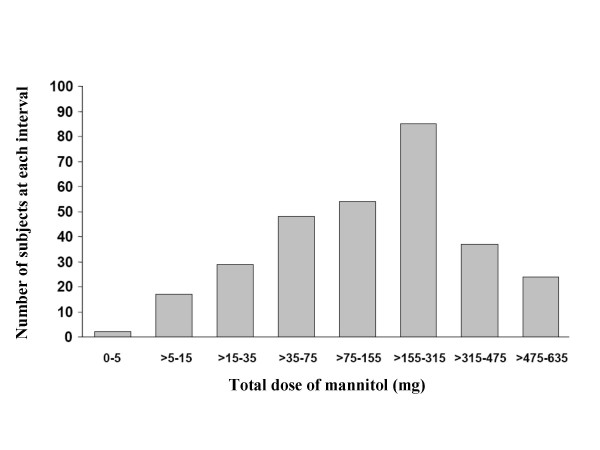
Number of subjects with a 15% fall in FEV_1 _to mannitol at each dose interval.

The highest proportion (28.7%) of subjects achieving a positive mannitol challenge result did so in the cumulative dose range of >155 × 315 mg, 18.2% were positive in the >75 × 155 dose range and 16.2% in the >35 × 75 dose range (Figure 3). Eighty percent of those who achieved a fall of 15% or more did so in a dose = 315 mg (10 capsules).

Nineteen (3.0%) subjects had repeat mannitol doses, 89.5% (n = 17) of whom went on to achieve a PD_15_, the remaining two subjects having maximum falls in FEV_1 _of 14.55% and 14.85%. Thirty-six recorded a PD_15 _to mannitol but did not achieve a PD_15 _to HS, while 62 achieved a PD_15 _to HS but not to mannitol. There was no significant sequence effect (p= 0.2) and no significant difference between the treatment order or the study site for mannitol and HS responses.

The mean ± SD time taken for a positive mannitol challenge was 17.3 ± 7.09 minutes and for HS was 15.0 ± 9.1 minutes. For a negative challenge the mean ± SD for mannitol was 26.4 ± 6.1 minutes and for HS it was 27.3 ± 3.6 minutes. It should be noted that this included the time taken to complete the post challenge measurement of the vital signs.

For subjects with a positive challenge test, the time to recover to 95% baseline FEV_1 _was similar for both mannitol and HS, being 19.4 ± 8.8 minutes and 19.3 ± 8.7 minutes respectively. The maximum recovery time was 65 minutes for mannitol and 79 minutes for HS. There were 344 (54.9%) subjects who received 200 mcg of salbutamol after the mannitol challenge, and 370 (58.2%) after HS. A second salbutamol dose of 200 mcg was given to 46 (7.3%) subjects after mannitol and 38 (6.0%) after HS, with additional bronchodilator given in 6 (1.0%) subjects following the mannitol challenge and 2 (0.3%) following HS.

There were 535 (85.3%) subjects who experienced cough during the mannitol challenge and 468 (73.5%) during the HS challenge. The mean cumulative dose at which cough was first reported was 62.7 mg for mannitol and 5.07 ml for HS. The majority of subjects, 70.8% for mannitol and 64.8% for HS, experienced occasional cough causing no delay in administering the next dose. Frequent cough causing a delay in the challenge was noted in 83 (13.2%) subjects during the mannitol challenge and 51 (8%) subjects during HS. There were 8 (1.3%) subjects during mannitol and 5 (0.8%) during HS who were noted to have a cough severe enough to stop the challenge although 2 and 4 respectively did record a 15% fall in FEV_1 _at the time they were stopped.

There were 14 (2.2%) subjects who commenced but did not complete the mannitol challenge and 9 (1.4%) subjects who did not complete the HS challenge. The most frequent causes of an incomplete challenge were incorrect calculation of the target 15% reduction in FEV_1 _(which resulted in premature termination of the challenge) and cough (see above).

The sensitivity and specificity for mannitol to identify responsiveness to HS and for the clinical diagnosis are given in Table 3. As expected the sensitivity for mannitol to identify a clinical diagnosis of asthma was lower due to many asthmatics taking ICS, however the specificity remained high. The sensitivity in children (<12 yrs) was 85.5% (95% CI: 76.1, 94.8) and the specificity was 68.4% (95% CI: 47.5, 79.0).

**Table 3:** Sensitivity and specificity of response to mannitol compared to hypertonic saline and a clinical assessment of asthma (Clinical Dx)

	Sensitivity (95% CI)	Specificity (95% CI)
Mannitol vs Hypertonic Saline	80.7 (76.4, 85.1)	86.7 (82.6, 90.7)
Mannitol vs Clinical Dx	59.8 (55.4, 64.2)	94.5 (89.9, 99.2)
Hypertonic Saline vs Clinical Dx	65.1 (60.9, 69.3)	95.2 (91.1, 99.3)
*Excluding all taking ICS*		
Mannitol vs Clinical Dx	70 (62.1, 78.2)	95 (90.7, 99.3)
*Excluding M-ve taking ICS*		
Mannitol vs Clinical Dx	89 (85.3, 92.1)	95 (90.7, 99.3)

Subjects diagnosed with asthma who were using ICS (either alone or in combination with a beta_2 _agonist) were divided into mannitol positive and negative. Of the 291 subjects mannitol positive, 204 (70.1 %) had been using ICS prior to the first challenge; of the 196 subjects negative to mannitol, 159 (81.1%) had been using ICS prior to the first challenge. As an exploratory analysis, the subjects who were mannitol negative and taking ICS before the study were removed from the analysis of sensitivity and specificity for mannitol with respect to the clinical assessment and there was an improvement in sensitivity (88.7%) and specificity (95.0%).

### Adverse events

In the 7 days following each challenge, 45.3% (n = 284) reported adverse events after mannitol and 44.7% (n = 284) after the HS. Applying the MedDRA classifications (Table 4), the nervous system was most frequently affected, followed by in decreased order of incidence; respiratory, thoracic and mediastinal disorders, gastrointestinal disorders, infections and infestations. There was no statistically significant difference in the incidence between these events for either challenge (p = ns). The most common related events in the 7 days following the mannitol challenge were headache, reported in 8.3% (n = 52), followed by pharyngolaryngeal pain in 3.5% (n = 22) and nausea in 2.4% (n = 15) of subjects. Headache was more prevalent in those negative to mannitol (13.6%) compared to those with a positive mannitol challenge (3.0%).

**Table 4:** Adverse events (AE) starting on the same day & between 1 & 7 days after challenge by MedDRA System Organ Class & Preferred Term (Safety population)

System Organ Class	Preferred Term	AE starting on same day as		AE starting between 1 & 7 days after	
		Mannitol challenge	Hypertonic Saline challenge	Mannitol challenge	Hypertonic Saline challenge

n		627 (%)	636 (%)	627 (%)	636 (%)
Any		102 (16.3)	84 (13.2)	217 (34.6)	228 (35.8)
Eye disorders		3 (0.5)	1 (0.2)	8 (1.3)	5 (0.8)
Gastrointestinal disorders		15 (2.4)	15 (2.4)	46 (7.3)	29 (4.6)
	Abdominal pain upper	1 (0.2)	3 (0.5)	11 (1.8)	6 (0.9)
	Diarrhoea NOS	1 (0.2)	1 (0.2)	7 (1.1)	3 (0.5)
	Nausea	7 (1.1)	9 (1.4)	20 (3.2)	9 (1.4)
General disorders and administration		10 (1.6)	6 (0.9)	22 (3.5)	13 (2.0)
Infections and infestations		2 (0.3)	4 (0.6)	26 (4.1)	40 (6.3)
	Nasopharyngitis	1 (0.2)	3 (0.5)	8 (1.3)	16 (2.5)
	Upper respiratory tract infect	1 (0.2)		6 (1.0)	11 (1.7)
Injury, poisoning and procedural complications			3 (0.5)	14 (2.2)	11 (1.7)
Investigations		4 (0.6)	4 (0.6)	1 (0.2)	2 (0.3)
Metabolism and nutrition disorders			1 (0.2)	2 (0.3)	
Musculoskeletal and connective tissue disorders		1 (0.2)	3 (0.5)	24 (3.8)	14 (2.2)
	Back pain		1 (0.2)	6 (1.0)	3 (0.5)
Nervous system disorders		46 (7.3)	38 (6.0)	87 (13.9)	101 (15.9)
	Headache NOS	38 (6.1)	32 (5.0)	78 (12.4)	92 (14.5)
Respiratory, thoracic and mediastinal disorders		40 (6.4)	21 (3.3)	48 (7.7)	61 (9.6)
	Asthma aggravated	1 (0.2)	3 (0.5)	6 (1.0)	5 (0.8)
	Cough	8 (1.3)	6 (0.9)	6 (1.0)	8 (1.3)
	Pharyngolaryngeal pain	16 (2.6)	5 (0.8)	16 (2.6)	13 (2.0)
	Rhinorrhoea	4 (0.6)		9 (1.4)	9 (1.4)
	Throat irritation	7 (1.1)		1 (0.2)	1 (0.2)
Skin and subcutaneous tissue disorders		1 (0.2)	4 (0.6)	10 (1.6)	11 (1.7)

Severe adverse events as defined by extremely distressed or unable to do usual activities were reported in 6.2% (n = 39) after mannitol and 4.7% (n = 30) after HS. The most common were respiratory, thoracic and mediastinal disorders reported in 2.1% (n = 13) after the mannitol and 1.4% (n = 9) after HS. Nervous system disorders were reported in 1.6% (n = 10) after mannitol and 1.9% (n = 12) after HS. These were headache in 1.3% (n = 8) after mannitol, and 1.1% (n = 7) after HS, followed by cough or aggravated cough in 0.8% (n = 5) after mannitol, and 0.3% (n = 2) after HS.

Withdrawal from the study due to adverse events occurred in four (0.6%) after the mannitol, and three (0.5%) after the HS. There were no statistically significant carryover effects for any of the baseline, end challenge, or recovery values for systolic and diastolic blood pressure, oxygen saturation, heart rate, and respiratory rate, either at the end of challenge or at recovery (p > 0.2) (Table 5).

**Table 5:** Vital signs at baseline (before pre challenge spirometry), and the change (Δ) at end of challenge and during recovery for the safety population

**Parameter**	**Challenge Point**	**Mannitol**	**Hypertonic Saline**
n		627	636
Heart Rate (beats/ minute)	Baseline	75.9 ± 13.4	76.2 ± 13.6
	End Challenge	Δ7.2 ± 10.8	Δ5.4 ± 9.2
	Recovery to pre Challenge FEV_1 _or 15 min post Challenge	Δ1.6 ± 10.0	Δ0.7 ± 9.3
Respiration Rate (per min)	Baseline	17.1 ± 4.3	17.1 ± 4.3
	End Challenge	Δ1.0 ± 3.3	Δ1.1 ± 3.7
	Recovery to pre Challenge FEV_1 _or 15 min post Challenge	Δ0.1 ± 2.9	Δ0.2 ± 3.0
Systolic Blood Pressure	Baseline	117.0 ± 14.8	116.9 ± 14.6
mm Hg	End Challenge	Δ1.5 ± 9.5	Δ1.5 ± 9.2
	Recovery to pre Challenge FEV_1 _or 15 min post Challenge	Δ-0.1 ± 8.8	Δ0.5 ± 9.1
Diastolic Blood Pressure	Baseline	72.0 ± 10.0	72.6 ± 10.2
mmHg	End Challenge	Δ2.4 ± 7.5	Δ1.7 ± 7.4
	Recovery to pre Challenge FEV_1 _or 15 min post Challenge	Δ1.2 ± 7.0	Δ0.4 ± 7.3
% oxygen saturation by	Baseline	97.2 ± 1.8	97.5 ± 1.6
pulse oximeter	End Challenge	Δ0.8 ± 2.0	Δ-0.7 ± 1.9
	Recovery to pre Challenge FEV_1 _or 15 min post Challenge	Δ-0.4 ± 1.6	Δ-0.1.4 ± 1.6

The incidence of respiratory symptoms as reported by symptom diary in the week following mannitol was very similar to that following HS and the duration and severity of these symptoms were very similar in both challenges (Table 6). There were 41.8% (n = 259) who experienced new or unusual symptoms in the week following the mannitol challenge, with a similar incidence, 41.1% (n = 257) after HS. Changes to existing medication regimens, or use of new medications were reported in the questionnaire in 26.7% (n = 165) after mannitol and 29.4% (n = 184) after HS.

**Table 6:** The incidence of respiratory symptoms as reported by symptom diary during 7 days following challenge for the safety population

**Symptom / beta**_2_**agonist use**	**Mannitol**	**Hypertonic Saline**
n	627 (%)	636 (%)
Daytime cough	352 (56.9)	365 (58.4)
Wheeze	270 (43.6)	280 (44.8)
Trouble breathing	270 (43.6)	262 (41.9)
Symptoms interfering with activities	172 (27.8)	178 (27.4)
Night time cough	212 (34.2)	236 (37.8)
Beta_2 _agonist use	319 (51.5)	333 (53.3)
Nebulised beta_2 _agonist	16 (2.6)	19 (3.0)

## Discussion

All asthmatics had an established history of asthma with good lung function that was similar to that seen with the non-asthmatics. Further, reliever bronchodilator was taken in 51.3% in the week prior to commencement of the study, however only 11.9% reported symptoms interfering with normal activity. The majority of adult asthmatic subjects, classified using GINA guidelines, had mild disease and most probably represent the population to be assessed using a bronchial provocation test, i.e. those with good lung function and mild symptoms.

The median doses of mannitol (148 mg) and HS (6.2 ml) to cause a 15% fall in FEV_1 _were similar to those that have previously been identified as the cut-off point between moderate and mild AHR to mannitol (155 mg) and HS (6.1 ml) [10]. Thus 50% of the asthmatic subjects were in the mild range of AHR to these agents and this was consistent with the cohort's treatment and symptom profile, i.e. symptoms less than once per week.

These results demonstrate the safety of the inhaled mannitol challenge to identify AHR. There were no serious adverse events recorded. There was no difference in the overall safety profile demonstrated by a similar adverse event profile between mannitol and HS. The low incidence of headache, the most frequent complaint after both challenges, was likely to be a direct result of the challenge procedure. Headache was more prevalent in those with a negative mannitol challenge and likely due to the larger number of forced expiratory manoeuvres required compared to those with a positive challenge.

The mean maximum fall in FEV_1 _was similar for both the mannitol and HS challenges and close to the target FEV_1 _of 15%. Relative to the potential fall in FEV_1 _that can occur following challenges such as exercise, this is a moderate decrease in FEV_1_. Few subjects had a fall in FEV_1 _greater than 30%, a value that is frequently attained when testing with exercise or with dry air as the stimulus [28,29]. Only two subjects responded positively to the initial mannitol dose of 5 mg and the falls in FEV_1 _were 15% and 26%. Twelve subjects had a PD_15 _to hypertonic saline = 1.0 ml which is equivalent to a 30 sec exposure to the aerosol.

Only two of the 19 subjects who had a between dose fall in FEV_1 _of >10% failed to achieve a PD_15 _and for both these subjects the maximum fall achieved was greater than 14.5%. Thus a fall in FEV_1 _of 10% between doses of mannitol should also be considered as a positive challenge and signal an end to the challenge procedure. The PD_15 _can then be extrapolated from the recorded response at the 10% fall.

Bronchoconstriction provoked by other challenge tests may be accompanied by significant reductions in SpO_2_, e.g. exercise challenge [28]. However the reduction in SpO_2 _during the mannitol challenge was small and was probably of clinical significance in only 3 subjects (i.e., >3%). There were only small and clinically insignificant changes in blood pressure, heart rate, and breathing frequency after challenge. This is in contrast to a challenge with exercise that can be associated with substantial changes in blood pressure, heart rate, and breathing frequency [28]. The insignificant changes in vital signs are relevant when choosing a test for the assessment of airway responsiveness in older subjects.

Cough is a recognised aspect of both the mannitol and HS challenges [30]. The majority of subjects (>85%) had either an occasional cough or no cough in response to the mannitol challenge. This finding is consistent with the mild severity of disease in the majority of asthmatic subjects and the high proportion taking ICS. Severe cough was infrequent, occurring in only 1.3% of subjects during the mannitol challenge. For 8 subjects the cough was too severe to continue the challenge.

The sensitivity of mannitol to identify subjects with AHR to HS was lower than expected, given the results of an earlier study [11]. The majority of subjects (52 of 62) who were negative to mannitol and positive to HS had AHR to HS within the mild range (PD_15_>6.1 ml). The reason for the discrepancy is not clear from the results but several explanations can be postulated. One explanation is that the HS is administered with tidal breathing whereas mannitol is given with an inhalation to total lung capacity. Cockcroft et al [31] have reported a similar discrepancy between methacholine responsiveness in mild subjects when the dosimeter technique that uses a deep inspiration is compared with the tidal breathing technique. Alternatively, it may relate to a higher than usual output of the nebulisers delivering the 4.5% saline. It would be expected that if too much fluid deposits in the airways it could amplify the airway narrowing effect of a small amount of smooth muscle contraction, thereby causing a false positive response [32]. Other possible reasons include subjects becoming more compliant with their medication after their initial challenge, and that the period between last medication and the challenge was variable, although a minimum of 12 hr was required.

It is likely that the relatively high number of negative challenges in subjects with asthma is largely due to the fact that such a high proportion of the subjects were using ICS and therefore their airway inflammation was well controlled. Airway sensitivity to mannitol is reduced or inhibited by treatment with ICS [17,18], a feature that allows it to be used for monitoring therapy [33]. The use of ICS largely explains the discrepancy between the sensitivity to both challenge tests and the clinical assessment, which was made on information that included medications taken by the subject. Of the total group of diagnosed asthmatic subjects, 75% were taking ICS.

Based on earlier studies, the sensitivity of the mannitol challenge would have been expected to be higher and, up to 95% in asthmatics not taking ICS [12]. Of the 196 subjects classified as asthmatic by a clinician and who were negative to mannitol, 81% (n = 159) were taking ICS. These subjects represented 33% of the 487 diagnosed asthmatic subjects. Re-analysis of the data after exclusion of this group showed a sensitivity of 88.7% for mannitol to identify a clinical diagnosis. This value is more consistent with findings in earlier studies. It should be noted however that use of the mannitol challenge for diagnostic purposes in subjects taking ICS warrants caution in the interpretation of a negative test result.

Koskela et al [18] have suggested that "if mannitol is used to monitor effect of ICS in asthma, the goal of treatment should be unresponsiveness". Further, Leuppi et al have used a negative test as a starting point for back titration of steroids [19]. Thus for those 70.1% of asthmatics taking ICS with a positive response to mannitol, the dose of ICS may have been inadequate and/or the compliance with its use poor so that the airway inflammation was still active.

In conclusion the mannitol challenge was generally safe and well tolerated. No serious adverse events were recorded. Mannitol PD_15 _had a sensitivity of 81% and specificity of 87% with respect to PD_15 _for 4.5% saline. A positive mannitol test cut off of a 15% fall in FEV_1 _(PD_15_) provided appropriate sensitivity and specificity with respect to clinical diagnosis of asthma even when the patient's baseline FEV_1 _was within the normal range. Based on an analysis of patients with a clinical diagnosis of asthma, and excluding those with a negative test result and on current corticosteroid therapy, mannitol PD_15 _had a sensitivity of up to 89% to detect the presence of asthma and specificity of 95% for clinical diagnosis of asthma.

### Declaration of competing interests

The study reported in this paper was sponsored by Pharmaxis Ltd. BC, AL and RF-M are shareholders, have options and are employees of Pharmaxis Ltd.

At the time of the study Dr Anderson, Dr Brannan, Ms Perry and Ms Freed-Martens were all employed, by the Central Sydney Area Health Service (now known as the Sydney South West Area Health Service) who owns the patents on the application described here for mannitol. All employees have benefited from the monies arising from the study in terms of attendance at scientific meetings but they have not received a personal financial benefit. Dr Anderson is the inventor named on the patent. The Area Health Service licensed the intellectual property to Pharmaxis Ltd in 2001.

The authors have no non-financial competing interests.

## Authors' contributions

All the authors contributed to the design of the protocol for this study. The data management and statistical analysis of the results was contracted out by Pharmaxis Ltd to Datapharm Australia Pty Ltd. This paper is a summary of their report and all the authors made a contribution to this summary.
